# Complicated intra-abdominal infections in a worldwide context: an observational prospective study (CIAOW Study)

**DOI:** 10.1186/1749-7922-8-1

**Published:** 2013-01-03

**Authors:** Massimo Sartelli, Fausto Catena, Luca Ansaloni, Ernest Moore, Mark Malangoni, George Velmahos, Raul Coimbra, Kaoru Koike, Ari Leppaniemi, Walter Biffl, Zsolt Balogh, Cino Bendinelli, Sanjay Gupta, Yoram Kluger, Ferdinando Agresta, Salomone Di Saverio, Gregorio Tugnoli, Elio Jovine, Carlos Ordonez, Carlos Augusto Gomes, Gerson Alves Pereira Junior, Kuo-Ching Yuan, Miklosh Bala, Miroslav P Peev, Yunfeng Cui, Sanjay Marwah, Sanoop Zachariah, Boris Sakakushev, Victor Kong, Adamu Ahmed, Ashraf Abbas, Ricardo Alessandro Teixeira Gonsaga, Gianluca Guercioni, Nereo Vettoretto, Elia Poiasina, Offir Ben-Ishay, Rafael Díaz-Nieto, Damien Massalou, Matej Skrovina, Ihor Gerych, Goran Augustin, Jakub Kenig, Vladimir Khokha, Cristian Tranà, Kenneth Yuh Yen Kok, Alain Chichom Mefire, Jae Gil Lee, Suk-Kyung Hong, Helmut Alfredo Segovia Lohse, Wagih Ghnnam, Alfredo Verni, Varut Lohsiriwat, Boonying Siribumrungwong, Alberto Tavares, Gianluca Baiocchi, Koray Das, Julien Jarry, Maurice Zida, Norio Sato, Kiyoshi Murata, Tomohisa Shoko, Takayuki Irahara, Ahmed O Hamedelneel, Noel Naidoo, Abdul Rashid Kayode Adesunkanmi, Yoshiro Kobe, AK Attri, Rajeev Sharma, Federico Coccolini, Tamer El Zalabany, Khalid Al Khalifa, Juan Sanjuan, Rita Barnabé, Wataru Ishii

**Affiliations:** 1 Department of Surgery, Macerata Hospital, Macerata, Italy; 2 Emergency Surgery, Maggiore Parma Hospital, Parma, Italy; 3 Department of General Surgery, Ospedali Riuniti, Bergamo, Italy; 4 Department of Surgery, Denver Health Medical Center, Denver, CO, USA; 5 American Board of Surgery, Philadelphia, PA, USA; 6 Harvard Medical School, Division of Trauma, Emergency Surgery and Surgical Critical Care Massachusetts General Hospital, Boston, MA, USA; 7 Department of Surgery, UC San Diego Health System, San Diego, CA, USA; 8 Department of Primary Care & Emergency Medicine, Kyoto University Graduate School of Medicine, Kyoto, Japan; 9 Department of Abdominal Surgery, University Hospital Meilahti, Helsinki, Finland; 10 Department of Surgery, University of Newcastle, Newcastle, NSW, Australia; 11 Department of Surgery, Govt Medical College and Hospital, Chandigarh, India; 12 Department of General Surgery, Rambam Health Care Campus, Haifa, Israel; 13 Department of Surgery, Adria Hospital, Adria, Italy; 14 Trauma Surgery Unit, Maggiore Hospital, Bologna, Italy; 15 Department of Surgery, Maggiore Hospital, Bologna, Italy; 16 Department of Surgery, Universidad del Valle, Fundacion Valle del Lili, Cali, Colombia; 17 Department of Surgery, Monte Sinai Hospital, Juiz de Fora, Brazil; 18 Emergency Unit, Department of Surgery, Ribeirão Preto, Brazil; 19 Department of Surgery, Chang Gung Memorial Hospital, Taoyuan, Taiwan; 20 Department of General Surgery, Hadassah Medical Center, Jerusalem, Israel; 21 Department of Surgery, Tianjin Nankai Hospital, Nankai Clinical School of Medicine, Tianjin Medical University, Tianjin, China; 22 Department of Surgery, Pt BDS Post-graduate Institute of Medical Sciences, Rohtak, India; 23 Department of Surgery, MOSC medical college, Cochin, India; 24 First Clinic of General Surgery, University Hospital /UMBAL/ St George Plovdiv, Plovdiv, Bulgaria; 25 General Surgery Clinic, Medical University/University Hospital St.George, Plovdiv, Bulgaria; 26 Department of Surgery, Edendale Hospital, Pietermaritzburg, Republic of South Africa; 27 Department of Surgery, Ahmadu Bello University Teaching Hospital Zaria, Kaduna, Nigeria; 28 Department of Surgery, Mansoura University Hospital, Mansoura, Egypt; 29 Department of Surgery, Faculdades Integradas Padre Albino, Catanduva, Brazil; 30 Department of Surgery, Mazzoni Hospital, Ascoli Piceno, Italy; 31 Department of Surgery, Mellini Hospital, Chiari (BS), Italy; 32 Department of General and Digestive Surgery, Virgen de la Victoria, University Hospital, Malaga, Spain; 33 Department of General Surgery and Surgical Oncology, Université de Nice Sophia-Antipolis, Universitary Hospital of Nice, Nice, France; 34 Department of Surgery, Hospital and Oncological Centre, Novy Jicin, Czech Republic; 35 Department of General Surgery, Lviv Emergency Hospital, Lviv, Ukraine; 36 Department of Surgery, University Hospital Center Zagreb, Zagreb, Croatia; 37 3rd Department of General Surger Jagiellonian Univeristy, Narutowicz Hospital, Krakow, Poland; 38 Department of Surgery, Mozyr City Hospital, Mozyr, Belarus; 39 Department of Surgery, Ancona University, Ancona, Italy; 40 Department of Surgery, Ripas Hospital, Bandar Seri Begawan, Brunei; 41 Clinical Sciences, Regional Hospitals Limbe and Buea, Limbe, Cameroon; 42 Department of Surgery, Severance Hospital, Yonsei University College of Medicine, Seoul, Republic of Korea; 43 Division of Trauma and Surgical Critical Care, Department of Surgery, University of Ulsan, Seoul, Republic of Korea; 44 II Cátedra de Clínica Quirúrgica, Hospital de Clínicas, Asuncion, Paraguay; 45 Department of Surgery, Khamis Mushayt General Hospital, Khamis Mushayt, Saudi Arabia; 46 Department of Surgery, Cutral Co Clinic, Neuquen, Argentina; 47 Department of Surgery, Faculty of Medicine Siriraj Hospital, Bangkok, Thailand; 48 Department of Surgery, Thammasat University Hospital, Pathumthani, Thailand; 49 Department of Surgery, Hospital Regional de Alta Especialidad del Bajio, Leon, Mexico; 50 Department of Clinical and Experimental Sciences, Brescia University, Brescia, Italy; 51 General Surgery, Adana Numune Training and Research Hospital, Adana, Turkey; 52 Visceral Surgery, Military Hospital Desgenettes, Lyon, France; 53 Visceral Surgery, Teaching Hospital Yalgado Ouedraogo, Ouagadougou, Burkina Faso; 54 Department of Acute and Critical care medicine, Tokyo Medical and Dental University, Tokyo, Japan; 55 The Shock Trauma and Emergency Medical Center, Matsudo City Hospital, Chiba, Japan; 56 Emergency and Critical Care Center of Nippon Medical School, Tama-Nagayama Hospital, Tokyo, Japan; 57 Department of Surgery, Our Lady of Lourdes Hospital, Drogheda, Ireland; 58 Department of Surgery, Port Shepstone Hospital, Port Shepstone, South Africa; 59 Department of Surgery, College of Health Sciences, Obafemi Awolowo University Hospital, Ile-Ife, Nigeria; 60 Department of Emergency and Critical Care Medicine, Chiba University Hospital, Chiba, Japan; 61 Department of Surgery, Bahrain Defence Force Hospital, Manama, Bahrain; 62 Department of Emergency Medicine, Kyoto Second Red Cross Hospital, Kyoto, Japan

## Abstract

Despite advances in diagnosis, surgery, and antimicrobial therapy, mortality rates associated with complicated intra-abdominal infections remain exceedingly high. The World Society of Emergency Surgery (WSES) has designed the CIAOW study in order to describe the clinical, microbiological, and management-related profiles of both community- and healthcare-acquired complicated intra-abdominal infections in a worldwide context. The CIAOW study (Complicated Intra-Abdominal infection Observational Worldwide Study) is a multicenter observational study currently underway in 57 medical institutions worldwide. The study includes patients undergoing surgery or interventional drainage to address complicated intra-abdominal infections. This preliminary report includes all data from almost the first two months of the six-month study period. Patients who met inclusion criteria with either community-acquired or healthcare-associated complicated intra-abdominal infections (IAIs) were included in the study. 702 patients with a mean age of 49.2 years (range 18–98) were enrolled in the study. 272 patients (38.7%) were women and 430 (62.3%) were men. Among these patients, 615 (87.6%) were affected by community-acquired IAIs while the remaining 87 (12.4%) suffered from healthcare-associated infections. Generalized peritonitis was observed in 304 patients (43.3%), whereas localized peritonitis or abscesses was registered in 398 (57.7%) patients.

The overall mortality rate was 10.1% (71/702). The final results of the CIAOW Study will be published following the conclusion of the study period in March 2013.

## Introduction

Intra-abdominal infections (IAIs) include a wide spectrum of pathological conditions, ranging from uncomplicated appendicitis to fecal peritonitis [1].

From a clinical perspective, IAIs are classified in two major categories: complicated and uncomplicated.

In uncomplicated IAIs, the infectious process only involves a single organ and does not spread to the peritoneum. Patients with such infections can be managed with either surgical resection or antibiotics. When the focus of infection is treated effectively by surgical excision, 24-hour perioperative prophylaxis is typically sufficient. Patients with less severe intra-abdominal infections, including acute diverticulitis and certain forms of acute appendicitis, may be treated non-operatively.

In complicated IAIs, the infectious process extends beyond a singularly affected organ, and causes either localized peritonitis or diffuse peritonitis. The treatment of patients with complicated intra-abdominal infections involves both source control and antibiotic therapy.

Intra-abdominal infections are further classified in two groups: community-acquired intra-abdominal infections (CA-IAIs) and healthcare-associated intra-abdominal infections (HA-IAIs). CA-IAIs are acquired directly in the community while HA-IAIs develop in hospitalized patients or residents of long-term healthcare facilities. HA-IAIs are associated with higher rates of mortality due to the patients’ poorer underlying health and an increased likelihood of infection by multi-drug resistant microorganisms.

Source control encompasses all measures undertaken to eliminate the source of infection and to control ongoing contamination.

The most common source of infection in community-acquired intra-abdominal infections is the appendix, followed by the colon, and then the stomach. Dehiscence complicates 5-10% of intra-abdominal bowel anastomoses and is associated with high rates of mortality [2].

Ultrasound- and CT-guided percutaneous drainage of abdominal and extra-peritoneal abscesses have proven to be safe and effective in select patients [3-10].

Surgery is the most important therapeutic recourse for controlling intra-abdominal infections.

Generally, the choice of the procedure depends on the anatomical source of infection, on the degree of peritoneal inflammation, on the generalized septic response and on the patient’s general conditions.

Patients suffering from severe peritonitis are prone to persisting intra-abdominal sepsis, even when the source of infection has been neutralized. Timely re-laparotomy is the only possible known surgical recourse, capable to significantly improve patient outcome in these cases.

In the event of secondary peritonitis, the decision and timing of re-laparotomy is largely subjective and is often based on a surgeon’s professional experience. Factors indicative of progressive or persistent organ failure during early postoperative follow-up analysis are the strongest indicators of ongoing infection and suggest positive findings upon re-laparotomy [11-13].

Three methods of localized, mechanical management of abdominal sepsis following the initial laparotomy, which was performed for purposes of source control, are currently debated within the medical community: open-abdomen, planned re-laparotomy and on-demand re-laparotomy

Antimicrobial therapy plays an integral role in the management of intra-abdominal infections, especially in critically ill patients requiring immediate empiric antibiotic therapy.

Empiric antibiotic therapy accounts for the most frequently isolated microorganisms as well as any local trends of antibiotic resistance.

The major pathogens involved in community-acquired intra-abdominal infections are Enterobacteriaceae and anaerobic microbes (especially B. fragilis).

An antimicrobial-based approach to treating intra-abdominal infections involves a delicate balance between the optimization of empirical therapy, which has been shown to improve clinical outcomes, and the reduction of excessive antimicrobial use, which has been proven to increase the rate of emergence of antimicrobial-resistant strains.

The threat of antimicrobial resistance is one of the major challenges associated with the antimicrobial management of complicated intra-abdominal infections.

The recent and rapid spread of serine carbapenemases in Klebsiella pneumoniae (KPC) has become an important concern when administering antimicrobial therapy in hospitals worldwide [14].

The growing emergence of multidrug-resistant bacteria and the limited availability of new antibiotics to counteract them has brought about an impending crisis with alarming implications (especially regarding gram-negative microorganisms).

## Methods

### Aim

The purpose of the study is to describe the clini-cal, microbiological, and treatment profiles of both community-associated and healthcare-acquired complicated intra-abdominal infections (IAIs) in a worldwide context.

Patients older than 18 years with both community-acquired and healthcare-associated intra-abdominal infections will be included in the database.

In Europe, the CIAO Study has recently ended, concluding a six-month, multicenter observational study across twenty European countries. The study’s findings have recently been published [15].

Given the promising results of the CIAO Study, the World Society of Emergency Surgery (WSES) has designed a prospective observational study investigating the management of complicated intra-abdominal infections in a worldwide context.

### Study population

The CIAOW study (Complicated Intra-Abdominal infection Observational Worldwide Study) is a multicenter observational study currently underway in 57 medical institutions worldwide. The study includes patients undergoing surgery or interventional drainage to address complicated IAIs.

**Figure 1:**
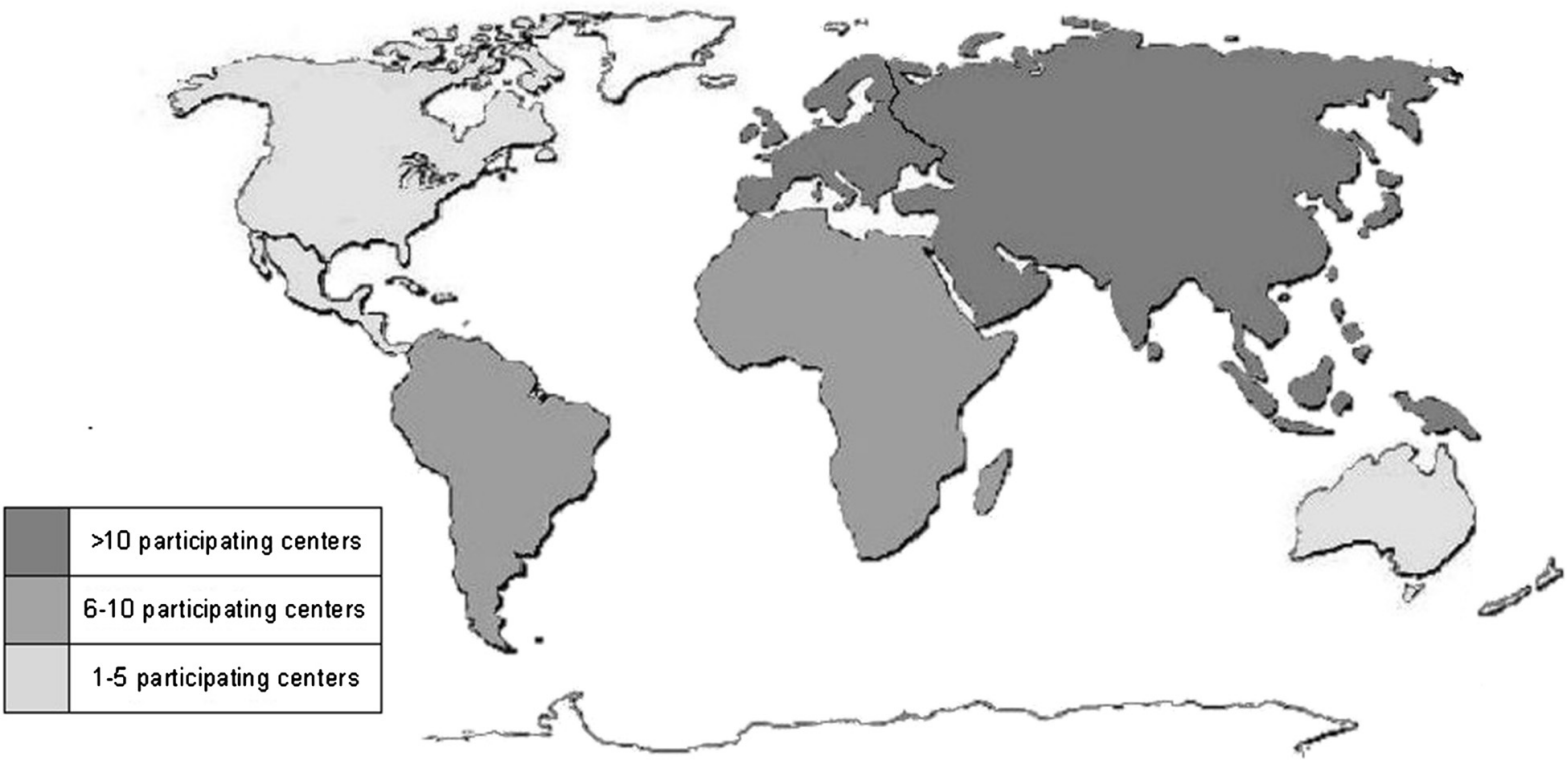
Participating centers for each continent.

Medical institutions from each continent participate in the study. The geographical distribution of the participating centers is represented in Figure 1.

### Study design

The study does not attempt to change or modify the laboratory or clinical practices of the participating physicians, and neither informed consent nor formal approval by an Ethics Committee has been required.

The study meets the standards outlined in the Declaration of Helsinki and Good Epidemiological Practices.

The study is monitored by the coordination center, which investigates and verifies missing or unclear data submitted to the central database. It is performed under the direct supervision of the board of directors of WSES.

### Data collection

In each center, the coordinator collects and compiles data in an online case report system.These data include the following: (i) patient and disease characteristics, i.e., demographic data, type of infection (community- or healthcare-acquired), severity criteria, previous curative antibiotic therapy administered in the 7 days preceding surgery; (ii) origin of infection and surgical procedures performed; and (iii) microbiological data, i.e., identification of bacteria and microbial pathogens within the peritoneal fluid, the presence of yeasts (if applicable), and the antibiotic susceptibilities of bacterial isolates.

The primary endpoints include the following:

•Clinical profiles of intra-abdominal infections

•Epidemiological profiles (epidemiology of the microorganisms isolated from intra-abdominal samples and these organisms’ resistance to antibiotics)

•Management profiles

### Statistical analysis

At the end of the six-month study period statistical comparisons will be performed using the Student’s t-test, χ2 analysis, or the Kruskall–Wallis/Wilcoxon tests, as dictated by the natural parameters of the data. Statistical significance will be defined as a P-value less than 0.05 (P < 0.05). Multivariate analysis will be carried out by means of stepwise logistic regressions in order to assess the predictive factors of mortality during hospitalization. Adjusted odds ratios (OR) and their 95% confidence intervals (CI) will also be included.

### Inclusion criteria

•Patients older than 18 years

•Community- and healthcare-acquired complicated intra-abdominal infections

### Exclusion criteria

•Age under 18 years old

•Pancreatitis

•Primary peritonitis.

## Preliminary results

### Patients

This preliminary report includes all data from the first two months of the six-month study period.

702 patients with a mean age of 49.2 years (range 18–98) were enrolled in the study. 272 patients (38.7%) were women and 430 (62.3%) were men. Among these patients, 615 (87.6%) were affected by community-acquired IAIs while the remaining 87 (12.4%) suffered from healthcare-associated infections.

304 patients (43.3%) were affected by generalized peritonitis while 398 (57.7%) suffered from localized peritonitis or abscesses.

112 patients (15.9%) were admitted in critical condition (severe sepsis, septic shock).

### Source control

The various sources of infection are outlined in Table 1. The most frequent source of infection was acute appendicitis. 243 cases were attributable to this condition.

**Table 1:** Source of infection

**Source of infection**	**Patients**
	**N 702 (100%)**
Appendicitis	243 (34.6%)
Cholecystitis	104 (14.8%)
Post-operative	53 (7.5%)
Colonic non diverticular perforation	38 (5.4%)
Gastroduodenal perforations	100 (14.2%)
Diverticulitis	40 (5.7%)
Small bowel perforation	53 (7.5%)
Others	52 (7.4%)
PID	8 (1.1%)
Post traumatic perforation	11 (1.6%)

The most frequently performed procedure employed to address complicated appendicitis was the open appendectomy. 136 patients (55.9%) admitted for complicated appendicitis underwent open appendectomies: 95 patients (69.8%) for localized infection or abscesses and 41 patients (31.2%) for generalized peritonitis. A laparoscopic appendectomy was performed on 93 patients (39.4%) presenting with complicated acute appendicitis, 82 (88.2%) and 11 (11.8%) of whom underwent the procedure for localized peritonitis/abscesses and generalized peritonitis, respectively. Open colonic resection was performed on 1 patient to address complicated appendicitis. In the other cases of complicated appendicitis, conservative treatment (percutaneous drainage, surgical drainage, and non-operative treatment) was performed. 7 (3%) patients underwent percutaneous drainage to address appendicular abscesses.

For patients with complicated acute cholecystitis (104 cases), the most frequently performed procedure to address cholecystitis was the open cholecystectomy. 53 cholecystitis patients (51%) underwent this procedure. A laparoscopic cholecystectomy was performed on 27 patients (26%). In the remaining cases, conservative treatment methods (percutaneous drainage, non-operative treatment) were alternatively employed.

Among the patients with complicated diverticulitis (40) the Hartmann resection was the most frequently performed procedure. 12 patients (30%) underwent a Hartmann resection. All these resections were open procedures. 8 of these patients underwent a Hartmann resection for generalized peritonitis, while the remaining 4 underwent the same procedure for localized peritonitis or abscesses.

Colo-rectal resection was performed in 11 cases (27.5%) (4 with and 7 without stoma protection).

The other patients received conservative treatment (percutaneous drainage, non-operative treatment, surgical drainage and stoma). Only two (5%) underwent laparoscopic lavage and drainage.

Of the 100 patients with gastro-duodenal perforations, the most frequent surgical procedure was gastro-duodenal suture. It was performed in 91 patients (91%): 85 patients underwent open gastro-duodenal suture and 6 patients underwent laparoscopic gastro-duodenal suture. Four (4%) patients underwent gastro-duodenal resection. The remaining patients (5%) received conservative treatment (non-operative treatment, surgical drainage).

Among the 53 patients with small bowel perforations, 35 underwent open small bowel resection (79.5%) and one (4.5%) underwent laparoscopic small bowel resection. Fourteen patients were treated by stoma. Two patients were treated by open drainage

Among the 38 patients with colonic non-diverticular perforation, 15 patients (66%) underwent open Hartmann resection, 1 patient (2.6%) underwent laparoscopic Hartmann resection, 9 (25%) underwent open resection with anastomosis and without stoma protection, and 4 underwent open resection with stoma protection (10.5%).

### Microbiology

Intraperitoneal specimens were collected from 415 (59.1%) patients.

Intraperitoneal specimens were isolated from 336 of the 615 patients with community-acquired intra-abdominal infections (54.6%). Among the remaining 87 patients with healthcare-associated intra-abdominal infections, intraperitoneal specimens were collected from 79 patients (90.9%).

The major pathogens involved in intra-abdominal infections were found to be Enterobacteriaceae.

The aerobic bacteria identified in samples of peritoneal fluid are reported in Table 2.

**Table 2:** Aerobic bacteria identified in peritoneal fluid

**Total**	**455 (100%)**
**Aerobic gram-negative bacteria**	**352**
Escherichia coli	226(49.7%)
(Escherichia coli resistant to third generation cephalosporins)	37 (8.1%)
Klebsiella pneuumoniae	53 (11.6%)
(Klebsiella pneumoniae resistant to third generation cephalosporins)	13 (2.9%)
Klebsiella oxytoca	3 (0.7%)
Enterobacter	10 (2.2%)
Proteus	13 (2.9%)
Pseudomonas	25 (5.5%)
Others	22 (4.8%)
**Aerobic gram-positive bacteria**	**103**
Enterococcus faecalis	27 (5.9%)
Enterococcus faecium	21 (4.6%)
Staphylococcus Aureus	11 (2.4%)
Streptococcus spp.	29 (6.5%)
Others	15 (3.3%)

According to CIAOW Study data, ESBL producers were the most commonly identified drug-resistant microorganism involved in IAIs.

1 identified isolate of Klebsiella pneumoniae proved resistant to Carbapenems.

Among the identified aerobic gram-negative isolates, there were 25 isolates of Pseudomonas aeruginosa, comprising 5.5% of all identified aerobic bacteria isolates.

Among the identified aerobic gram-positive bacteria, Enterococci (E. faecalis and E. faecium) were the most prevalent, representing 10.5% of all aerobic isolates, 3 glycopeptide-resistant Enterococci were identified; 2 were glycopeptide-resistant Enterococcus faecalis isolates and 1 was glycopeptide-resistant Enterococcus faecium isolates.

Tests for anaerobes were conducted for 168 patients.

52 anaerobes were observed. The most frequently identified anaerobic pathogen was Bacteroides. 39 Bacteroides isolates were observed during the course of the study.

Additionally, 36 Candida isolates were collectively identified. 30 were Candida albicans and 6 were non-albicans Candida.

### Outcome

The overall mortality rate was 10.1% (71/702).

68 patients (9.7%) were admitted to the intensive care unit in the early recovery phase immediately following surgery.

90 patients (12.8%) ultimately required additional surgeries; 54% of these underwent relaparotomies “on-demand”, 28.9% underwent open abdomen procedures.

According to univariate statistical analysis, a critical clinical condition (severe sepsis and septic shock) upon hospital admission was the most significant risk factor for death; indeed, the rate of patient mortality was 36.6% (41/112) among critically ill patients (patients presenting with septic shock and severe sepsis upon admission), but the mortality rate was only 5.1% (30/590) for clinically stable patients (p < 0.0001).

For patients with generalized peritonitis, the mortality rate was 18% (55/304) while patients with localized peritonitis or abscesses demonstrated a mortality rate of only 4% (16/398) (p < 0,001).

The immediate post-operative clinical course was a significant parameter for predicting mortality: the rate of patient mortality was 54.9% (51/93) among critically ill patients (patients presenting with septic shock and severe sepsis upon the immediate post-operative course), but the mortality rate was only 3.3% (20/609) for clinically stable patients (p < 0.0001).

Preliminary statistical analyses were performed using MedCalc^®^ statistical software.

## Conclusion

Complicated intra-abdominal infections remain an important cause of morbidity with poor clinical prognoses.

The purpose of the CIAOW Study is to describe the epidemiological, clinical, microbiological, and treatment profiles of both community-acquired and healthcare-acquired complicated intra-abdominal infections (IAIs) based on the data collected over a six-month period (October 2012 to March 2013) from 56 medical institutions Worldwide.

The final results of the CIAOW Study will be published following the conclusion of the study period in March 2013.

## Competing interests

The authors declare that they have no competing interests.

## Authors’ contributions

MS designed the study and wrote the manuscript. All authors read and approved the final manuscript.
